# Natural killer cells in combination with the inhibition of telomerase induced apoptosis in Acute Myeloid Leukemia cells

**DOI:** 10.1016/j.bbrep.2025.102027

**Published:** 2025-04-26

**Authors:** Ali Rafat, Khadijeh Dizaji Asl, Zeinab Mazloumi, Mehdi Talebi, Hojjatollah Nozad Charoudeh

**Affiliations:** a Student Research Committee, Tabriz University of Medical Sciences, Tabriz, Iran; b Anatomical Sciences Research Center, Institute for Basic Sciences, Kashan University of Medical Sciences, Kashan, Iran; c Department of Histopathology and Anatomy, Faculty of Medical Sciences, Tabriz Medical Sciences, Islamic Azad University, Tabriz, Iran; d Department of Applied Cell Sciences, Faculty of Advanced Medical Sciences, Tabriz University of Medical Sciences, Tabriz, Iran; e Stem Cell Research Center, Tabriz University of Medical Sciences, Tabriz, Iran; f Scientific Center of Zoology and Hydroecology, NAS RA, Yerevan, Armenia

**Keywords:** Acute myeloid leukemia, Apoptosis, Natural killer cell, Telomerase

## Abstract

**Background:**

Recent trends in developing new treatments for cancers, highlight the use of immune cells particularly Natural Killer (NK) cells, as promising therapeutic strategies. While NK cells exhibit significant anti-tumor effects, their effectiveness is often limited. This study investigated the impact of BIBR1532, a human telomerase reverse transcriptase (hTERT) inhibitor, on improving the cytotoxicity of NK cells against Acute Myeloid Leukemia (AML) cells.

**Methods:**

Primary AML cells and Kg-1a cell lines were cultured and treated with the half-maximal inhibitory concentration (IC50) of BIBR1532 for 48 h. The treated cells were then co-cultured with NK cells, after which cytotoxicity, cell proliferation, and apoptosis were assessed using Annexin V/7-AAD and Ki-67 expression analysis. Finally, apoptosis-related genes and proteins, hTERT gene and caspase 3/7 activity were studied.

**Results:**

The Telomerase Inhibition (TI) in primary AML and Kg-1a cells with IC50 values of 38.75 μM and 57.64 μM, respectively, sensitized the AML cells and enhanced the anti-proliferative effects of NK cells. The combination of BIBR1532 and NK cells led to increased apoptosis, as indicated by the upregulation of the Bax and Bad genes, an increased Bax/Bcl-2 ratio, caspase 3/7 activity, Bax protein and a downregulation of mRNA expression levels of Bcl-2, Bcl-xl and decreased Bcl-2 protein.

**Conclusion:**

The findings of this study demonstrate that the concurrent application of BIBR1532 and NK cells promotes apoptosis and reduces proliferation by targeting apoptosis-related genes and proteins such as Bax and Bcl-2.

## Highlights

• Telomerase inhibition sensitizes AML cells in treatment with NK cells.
• Inhibition of telomerase increases NK cells cytotoxic activity against AML cells.
• Telomerase inhibitor with NK cells boosts apoptosis in AML cells over mono-therapy.

## Graphical abstract

**Figure:**
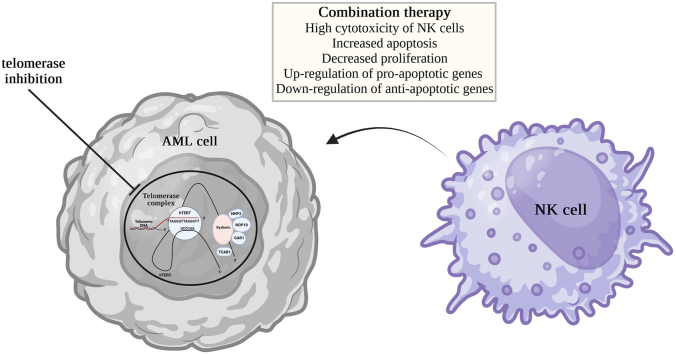

## 1 Introduction

For decades, strategies such as surgery, chemotherapeutic agents, and radiotherapy have been utilized to treat malignancies. however, resistance to chemotherapy and radiotherapy significantly contributes to cancer relapse. Many studies have focused on effective approaches to overcome tumor cell resistance by using immune cells such as Natural Killer (NK) cells. These cells are key members of innate immunity which actively controls the viral infections and prevent neoplastic development via a process known as tumor immunosurveillance [1]. The use of NK cells is an attractive strategy for adoptive immunotherapy. These cells can fight cancer cells without requiring Major Histocompatibility Complex (MHC) restriction, recognition, or prior immunization [2]. NK cells stimulate rapid and short-term responses by releasing different cytokines and triggering adaptive immune responses against cancer cells [3,4]. Owing to the advances in NK cell studies, NK cell-based immunotherapy is being has emerged as a new antitumor therapy for many types of neoplasia [5], including Acute Myeloid Leukemia (AML) [6,7].

AML is a fast-progressing lethal blood cancer characterized by the accumulation of malignant blasts in bone marrow [8]. The disorder is initiated and maintained by a rare population of Leukemia Stem Cells (LSCs) that acquire properties of cell cycle quiescence, self-renewal, and chemoresistance [4,9]. In AML patients, the function of NK cells is inhibited, allowing cancer cells to evade being detected by the immune system. NK cell immunotherapy aims to reactivate NK cells, enabling them to effectively target and kill cancer cells. The ongoing clinical trials are scrutinizing different approaches to NK cell-based immunotherapies, such as Cytokine-Induced Memory-Like (CIML) NK cells, Chimeric Antigen Receptor (CAR) NK cells, and adoptive NK cell transfer [10,11]. However, relying solely on NK cells has shown limited efficacy, despite their significant antitumor properties [12]. Therefore, along with enhancing the effect of NK cells, while developing clinical methods with minimal side effects.

In the present study, the co-treatment effects of NK cells and telomerase inhibitor (BIBR1532) were evaluated on AML cells. Telomerase, a specialized ribonucleoprotein DNA polymerase enzyme, has two major subunits and several accessory proteins [13,14]. Two subunits consist of (1) human Telomerase Reverse Transcriptase (hTERT) and (2) human Telomerase RNA Component (hTERC). By maintaining telomere length, telomerase ensures genome stability in immune cells, stem cells, and cancer cells [15]. BIBR1532 is a highly selective non-peptide and non-nucleoside small molecule telomerase inhibitor. It non-competitively binds to the hTERT subunit of telomerase and inhibits its function [16,17]. BIBR1532 induces apoptosis and inhibits proliferation in leukemia cells without affecting normal hematopoietic stem cells [18,19]. The anti-cancer effects of telomerase inhibition by BIBR1532 have been reported in several cancers such as leukemia, lung, breast, ovarian, and chondrosarcoma [14,20,21]. Furthermore, telomerase inhibition not only acts as an effective anti-proliferative agent but also sensitizes cancer cells to other anti-cancer agents [20]. Given the role of BIBR1532, this inhibitor shows promise for improving treatment strategies in AML patients. In the previous study, the effects of telomerase inhibitor and immunotherapy were combined to improve the cytotoxic effects of immunotherapy against breast cancer cells and enhance therapeutic potential. It was shown that telomerase inhibition, using BIBR1532, enhanced cancer cell susceptibility to NK cell therapy [22]. In the present study, co-treatment of telomerase inhibition and NK cell cytotoxicity on Kg-1a and primary AML cells was evaluated. The findings supported the administration of a combined approach involving telomerase inhibition and immunotherapy as a viable treatment strategy for AML.

## 2 Materials and methods

### 2.1 Samples, cell culture, blood mononuclear collection, and CD34^+^ enrichment

Blood samples (7 males and 3 females) were collected from the posterior iliac crest of newly diagnosed AML patients, with a mean age of 63 years (ranging from 59 to 65 years). The samples were placed in sterile tubes containing heparin after obtaining written consent in accordance with the Declaration of Helsinki. Ethical approval for this study was obtained from the ethics committee of Tabriz University of Medical Sciences (TUOMS) (ethics code: IR.TBZMED.REC.1397.1041). Each patient's blood sample (averaging 20 ml) was diluted 1:2 in Phosphate Buffer Saline (PBS) supplemented with 5 % Fetal Bovine Serum (FBS [Gibco]). Blood mononuclear cells (BMCs) were isolated using Ficoll (density = 1.077 g/ml; Innotrain, Germany) at 850 g (acceleration 4; no brake) for 25 min at 4 °C. The buffy coat was then collected and washed twice with PBS to eliminate any residual components. Cell viability was evaluated using Trypan blue staining with a hemocytometer. The Kg-1a cancer cell line was obtained from the National Cell Bank of Iran (Pasteur Institute, Tehran, Iran) and cultured in RPMI1640 medium supplemented with 20 % FBS and 1 % penicillin/streptomycin at 37 °C. The Magnetic-Activated Cell Sorting (MACS) system (Miltenyi Biotec, Berlin, Germany) was employed to enrich primary AML cells (CD34^+^ cells) as described by Rafat et al. [14].

### 2.2 CD56^+^ cells enrichment

Circulating NK cells were isolated from total Peripheral Blood Mononuclear Cells (PBMCs) obtained from healthy donors. CD56 NK cells were separated using a MACS system. PBMCs were first incubated with 100 μl of blocking antibody and anti-CD56 microbeads (Miltenyi Biotec, Berlin, Germany) for 30 min at 4 °C. The labeled cells were then passed through an LS column, and CD56^+^ cells were collected by flushing them into a 15 ml tube. The expression levels of CD56-positive cells were analyzed using flow cytometry with an anti-CD56 antibody (PE conjugated, Biolegend).

### 2.3 Telomerase inhibition

BIBR1532 (Cayman Chemical, MI, USA) was dissolved in 0.1 % sterile dimethyl sulfoxide (DMSO) to prepare a stock solution at a concentration of 10 mM, which was stored at −20 °C. Working concentrations of BIBR1532 were then prepared at 10, 30, 60, and 90 μM. A control group lacking both the inhibitor and DMSO was included in the experimental setup.

### 2.4 Cytotoxicity assays

The IC50 of BIBR1532 in primary AML and Kg-1a cells was determined using the MTT (3- [4, 5-dimethylthiazol-2-yl]-2, 5-diphenyl tetrazolium bromide) assay. Cells (5×10^4^) were incubated in each well, and BIBR1532 was added at the specified concentrations and incubated for 24, 48, and 72 h. After incubation, MTT solution (M6494/Sigma, 5 mg/ml in PBS) was added to each well, followed by a 4-h incubation at 37 °C. Each well was then supplemented with 100 μl of a solution containing 10 % Sodium Dodecyl Sulfate (SDS) with 0.1 % HCl and incubated overnight. The absorbance of each well was measured at 570 nm using a microplate reader (BioTek ELx 808, USA).

### 2.5 Cell proliferation assay

For intracellular staining, cells were permeabilized on ice for 20 min using the BD Cytofix/Cytoperm Plus Fixation/Permeabilization kit (BD Biosciences, San Jose, CA) at ratio of 500 μl per 1 × 10∗6 cells. Following this, the cells were incubated with a Ki-67 antibody for 30 min, the cells were washed and resuspended in 500 μl of staining buffer. Samples were analyzed by FACS (BD Biosciences) and FlowJo software(version X.0.7).

### 2.6 NK cell function assay

The cytotoxicity of NK cells was evaluated by measuring CD107a expression and IFN-γ secretion. CD56^+^ NK cells were enriched from PBMCs of a healthy donor using MACS. NK cells (5 × 10^5 per well) were seeded in a 96-well plate with a volume of 100 μl of RPMI medium containing 10 % FBS and 1 % P/S. Approximately 1×10^5^ cells from Kg-1a or primary AML samples (T) were added to each well and co-cultured with effector NK cells (E) at an effector-to-target (E:T) ratio of 5:1 in a final volume of 200 μl. Subsequently, 10 μL of CD107a (LAMP-1) antibody (PE conjugated, Biolegend) was added, and the cells were incubated for 5 h at 37 °C in a 5 % CO_2_ environment. To assess IFN-γ (an intracellular marker), the cells were first permeabilized using the BD Cytofix/Cytoperm Plus Fixation/Permeabilization kit (BD Biosciences, San Jose, CA) at the ratio of 500 μl per 1 × 10^6^ cells on ice for 20 min. Following this, the cells were stained with PE conjugated IFN-γ antibody (clone: B27, BD Biosciences) (1.5 μl/1×10^6^ cells) at 4 °C in the dark for 30 min. The cells were then washed with 1 ml of 1x Perm Wash Buffer and analyzed using FACS (BD Biosciences). The results were processed using FlowJo software (X.0.7). For the positive control group, PMA (50 ng/200 μl) was used.

### 2.7 Caspase 3,7 activity assay

Caspase-3/7 activity was quantitatively measured using a colorimetric assay kit (Kiazist Life Sciences (KCAS37), Iran), following the manufacturer's protocol. In brief, primary AML and Kg-1a cells were seeded in 6-well plates at a density of 2×10^5^ cells per well. Following a 24 h incubation period with nanoparticles (NPs) at a concentration of 2.5 mg/mL, cells were harvested, enumerated, and lysed using 500 μL of caspase lysis buffer per 5×10^5^ cells. Cell lysis was achieved through a 20 min incubation at 4 °C, followed by centrifugation at 12,000 rpm for 15 min at 4 °C. Supernatants were collected and stored at −80 °C until analysis. For the assay, 50 μL of cell lysate was mixed with 50 μL of caspase buffer, 0.5 μL of dithiothreitol (DTT), and 5 μL of caspase substrate in each well of a 96-well plate. Following a 5 h incubation at 37 °C, absorbance was measured at 405 nm using a plate reader. Caspase-3/7 activity was determined by referencing a standard curve generated from kit-provided standards.

### 2.8 Apoptosis assay

Initially, the IC50 values of BIBR1532 were applied to primary AML and Kg-1a cells. After two days, the AML cells were washed and co-cultured with isolated NK cells at an Effector to Target (E:T) ratio of 5:1, as previously described. Following incubation, the cell mixtures were collected and centrifuged for 5 min at 300 g. To evaluate apoptosis, the Annexin-V/7AAD kit was employed. Briefly, the cells were resuspended in 100 μL of 1X binding buffer (BioLegend). Then, 5 μL of annexin V antibody and 5 μL of 7AAD were added, and the mixture was incubated in the dark on ice for 25 min. The cells were then suspended in 500 μL of binding buffer and analyzed using FACS (BD Biosciences) and FlowJo software (version X.0.7).

### 2.9 Assessment of Bax and Bcl-2 proteins by ELISA assay

The protein levels of Bcl-2 and Bax were assessed using an enzyme-linked immunosorbent assay (ELISA) following the treatment of primary AML and Kg-1a cells with BIBR1532 and NK cells. Micro-ELISA plates were pre-coated with antibodies specific to Human B-cell leukemia (Bcl-2, Elabscience, EEL-H0114) and Human Bcl-2 Associated X Protein (Bax, Elabscience, E-EL-H0562). In accordance with the manufacturer's instructions, 100 μL of samples or standards was added to the wells of the micro-ELISA plate and incubated for 90 min at 37 °C to allow binding to the coated antibodies. Subsequently, 100 μL of a biotinylated detection antibody specific to Human Bcl-2/Bax and 100 μL of an Avidin-Horseradish Peroxidase (HRP) conjugate were added in succession to each well, with incubation at 37 °C for 60 min and 30 min, respectively. Unbound components were removed by washing with wash buffer between incubation periods. Following this, 90 μL of substrate solution was added to each well, and the reaction was halted with the addition of a stop solution (50 μL) to each well. The resulting yellow color from the enzyme-substrate reaction was measured using a multilabel plate reader at a wavelength of 450 nm.

### 2.10 RNA extraction, cDNA synthesis, and RT-PCR amplification

Total RNA was extracted using a total RNA extraction kit accordance with the manufacturer's instructions (Yekta Tajhiz Azma, Iran). The quality of the RNA samples was evaluated spectrophotometrically using a NanoDrop 1000 spectrophotometer (Thermo Fisher Scientific). Complementary DNA synthesis was performed using the First Strand cDNA Synthesis kit (Yekta Tajhiz Azma, Iran). The Real-time PCR array consisted of 10 μL of SYBR Green qPCR master mix (2X) (Biofact, South Korea), 2 μL of cDNA product, 0.5 μL of each forward and reverse primer (10 pmol) as indicated in Table 1, and 7 μL of nuclease-free water, yielding a total volume of 20 μL per reaction. Data were analyzed using LightCycler® 96 system (Roche Diagnostics) to obtain CT values. For normalization, β-actin was used, and the data were processed using the 2-ΔΔCT method.

**Table 1:** Nucleotide sequences of the primers used for Real-Time RT-PCR.

Gene	Forward primer (5′–3′)	Reverse primer (5′–3′)	Size (bp)
Β-actin	AAACTGGAACGGTGAAGGTG	TATAGAGAAGTGGGGTGGCT	174
hTERT	CAGCAAGTTTGGAAGAACCC	GACATCCCTGCGTTCTTGG	234
Bax	TCACTGAAGCGACTGATGTCC	CTCCCGCCACAAAGATGGTC	194
Bad	ACTTCCTCGCCCGAAGAGC	CTTCCCCTGCCCAAGTTCC	198
Bcl-2	AGTGAACATTTCGGTGACTT	CTTCCAGACATTCGGAGACC	208
Bcl-xl	ATCCCAGCTCCACATCACC	CGATCCGACTCACCAATACC	202

### 2.11 Statistical analysis

Statistical analysis was conducted using GraphPad Prism version 7. The results are presented as Mean ± Standard Deviation (SD) from three independent assays. A one-way analysis of variance (ANOVA) was employed to determine significant differences between groups, followed by the Tukey post-hoc test to identify specific inter-group variations. P-values of ∗P < 0.05, ∗∗P < 0.01, ∗∗∗P < 0.001, and ∗∗∗∗P < 0.0001 were regarded as statistically significant.

## 3 Results

### 3.1 Telomerase inhibition by BIBR1532 decreased the viability of Acute Myeloid Leukemia cells in a time/concentration-dependent manner

**Fig. 1:**
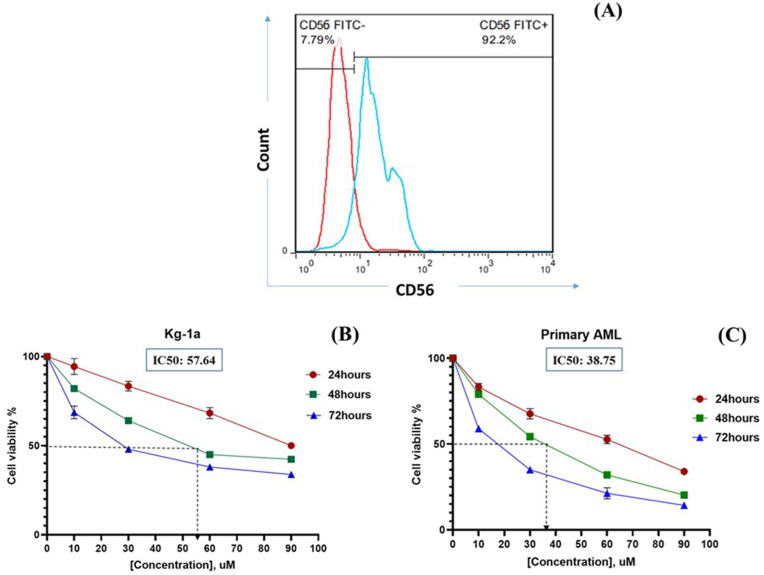
The percentage of CD56^+^ cells after double-positive enrichment by MACS (A). The effect of TI by BIBR1532 on cell viability of Kg-1a (B) and primary AML cells (C). Cells were incubated with different doses of BIBR1532 (10, 30, 60, and 90 μM) at 24, 48, and 72 h. IC50 values of Kg-1a and primary AML cells are illustrated. The values are given as mean ± SD of three independent experiments (P < 0.0001).

To achieve high purity of CD56^+^ cells, double enrichment was performed. The percentage of CD56^+^ cells were obtained through double enrichment process was 92.2 % (Fig. 1A). AML cells were incubated for 24, 48, and 72h with different concentrations of BIBR1532 (10, 30, 60, and 90 μM). According to Fig. 1A, BIBR1532 stopped the Kg-1a cell growth rate in a time/concentration-dependent manner, after 24, 48, and 72 h incubations. The incubation of Kg-1a cells with 90 μM BIBR1532 reduced cell viability to 58.45 % (24 h), 46.76 % (48 h), and 33.21 % (72 h) (Fig. 1B). As shown in Figs. 1C and 90 μM dosage decreased cell viability to 34.82 % (24 h), 20.56 % (48 h) and 14.98 % (72 h) when compared to the control group in the primary AML cells. IC50 value in the treatment of Kg-1a cells with BIBR1532 was approximately 87.21, 57.64, and 27.54 μM in 24, 48, and 72 h (Fig. 1B) while, in primary AML cells it was 64.35, 38.75, and 14.36 μM, respectively (Fig. 1C).

### 3.2 Cytotoxic activity of natural killer cells was increased against Acute Myeloid Leukemia cells after TI

**Fig. 2:**
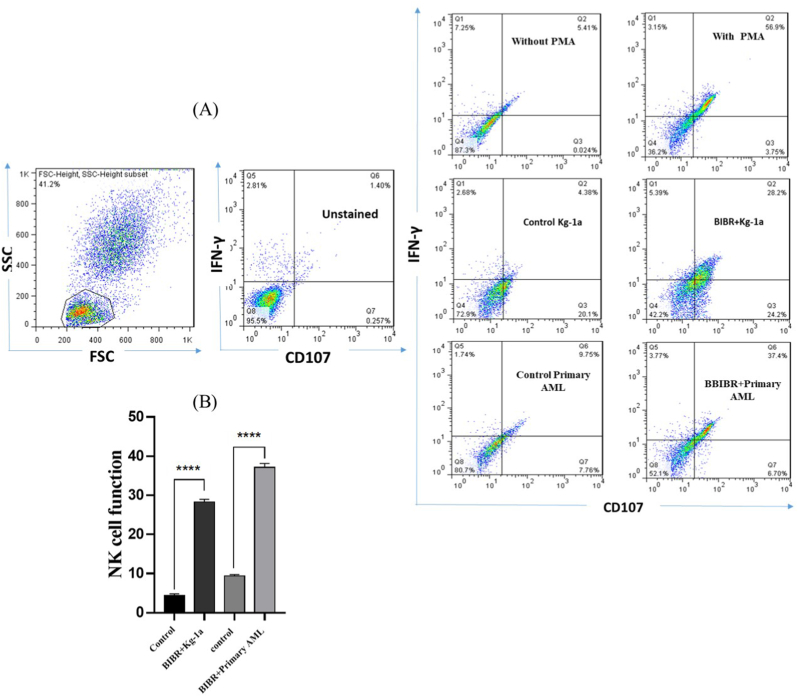
NK cells displayed sufficient cytotoxic activity against AML cells after TI. Representative flow cytometry profile of CD107a/IFN-γ expression on NK cells when incubated with Kg-1a and primary AML cells in an effector: target (E: T) ratio of 5:1 before (Control) and after TI (BIBR1532). The significant differences between the two groups assess by two-tailed Student's t-test. The values are shown as mean ± SD of 3 independent experiments, ∗∗∗P ≤ 0.0001.

To evaluate the NK cell activity before and after telomerase inhibition(TI) in Kg-1a and primary AML cells, IFN-γ/CD107-a staining was performed (Fig. 2). As shown in Fig. 2B, NK cell cytotoxic activity before TI on Kg-1a cells (control) was about 4 %; however, after TI on Kg-1a cells using BIBR1532, NK cell cytotoxic activity significantly increased to approximately 28 %. Also, the cytotoxic activity of NK cells before and after TI on primary AML cells was about 9.75 and 37.5 % respectively. NK cells exhibited high cytotoxic activity (IFN-γ/CD107-a) when telomerase was inhibited on Kg-1a and primary AML cells compared to the control group.

### 3.3 The combination of telomerase inhibition/natural killer cells significantly exerted anti-proliferative effects

**Fig. 3:**
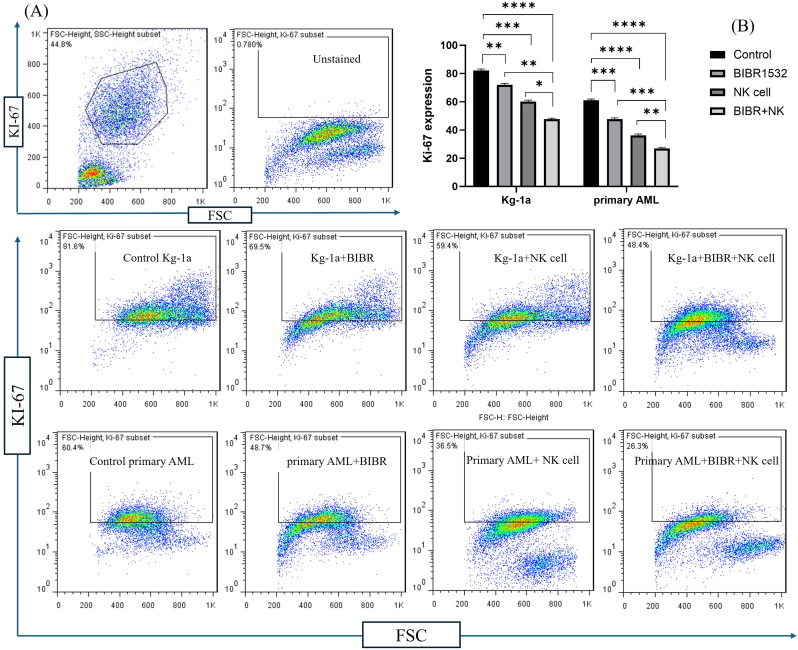
TI/NK cells co-treatment decreased cell proliferation in AML cells. The effect of NK cells and TI combination on cell proliferation rate was assessed using Ki-67 staining (A). Mono and combination treatment with TI and NK cells significantly decreased Ki-67 positive Kg-1a and primary AML cells when compared to the controls (without inhibitor and NK cells) (B). Moreover, Ki-67 positive cells significantly reduced after incubation with the combination of TI and NK cell when compared to the treatment with NK cells and TI in both Kg-1a and primary AML cells (B). The significant differences among groups assess by two‐way ANOVA. The values are shown as mean ± SD of 3 independent experiments, ∗P ≤ 0.05, ∗∗p < 0.01, ∗∗∗P ≤ 0.001 and ∗∗∗∗P ≤ 0.0001.

To investigate whether TI could enhance the anti-proliferative activity of NK cells in AML cells, the inhibitory effects of both single and combination treatments of NK cells and TI were evaluated on cell proliferation of Kg-1a and primary AML cells with Ki-67 staining. The percentage of Ki-67 positive Kg-1a and primary AML cells (control) before treatment were 82.1 % and 60.7 %, respectively (Fig. 3B). Monotherapy by TI and NK cells significantly reduced cell proliferation in Kg-1a cells to 71.4 % and 59.4 %, respectively. Moreover, co-treatment with TI/NK cells significantly reduced cell proliferation to 48.4 % compared to monotherapy with TI or NK cells. Also, cell proliferation following monotherapy with TI and NK cells decreased in primary AML cells and reached 46.2 % and 36.5 % respectively. Furthermore, the combination therapy with TI/NK cells significantly decreased cell proliferation to 26.3 % compared to monotherapy with TI and NK cells. These findings suggest that TI enhanced the anti-proliferative effect of NK cells in AML cells.

### 3.4 Telomerase inhibition enhanced natural killer cell-induced apoptosis in Acute Myeloid Leukemia cells

**Fig. 4:**
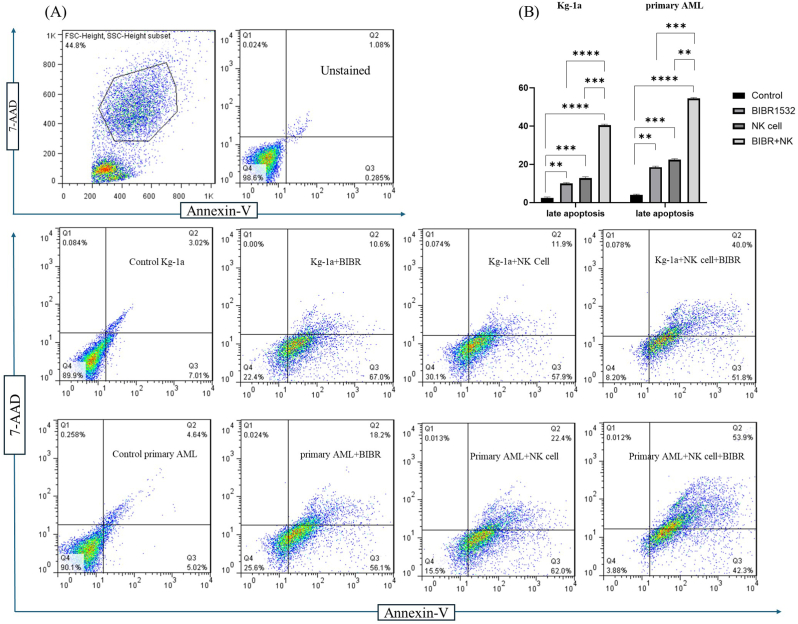
TI enhanced NK cell-induced apoptosis in AML cells. After monotherapy with TI and NK cells and co-treatment with TI and NK cells in Kg-1a and primary AML cells, Annexin-V+/7AAD+ (late apoptosis) cells percentage significantly increased compared to the controls (no treatment) (B). Annexin-V+/7AAD+ (late apoptosis) cells percentage significantly increased in response to co-treatment with the NK cell and TI for 48 h in comparison to monotherapy (BIBR1532 and NK cell) in both Kg-1a and primary AML cells (B). The Annexin-V-/7AAD- (Q4) showed viable cells, The Annexin-V+/7AAD- (Q3) showed early apoptotic cells and Annexin-V+/7AAD+ (Q2) show apoptotic cells respectively. The significant differences among groups assess by two‐way ANOVA. The values are shown as mean ± (SD) of 3 independent experiments, ∗∗p < 0.01, ∗∗∗P ≤ 0.001, and ∗∗∗∗P ≤ 0.0001.

Annexin-V/7AAD staining was performed to assess whether telomerase inhibition enhances NK cell-induced cytotoxicity. Although NK cells and TI separately enhanced apoptotic cell death, their combination treatment amplified the percentage of late apoptosis (Annexin-V+/7AAD+) in AML cells (Fig. 4A). As shown in Fig. 4B, the percentage of late apoptosis cells was approximately 40 % in the co-treatment group, compared to 11.9 % in NK cell-treated cells and 8.3 % in TI treated cells. Similarly, in primary AML cells, the percentage of late apoptosis cells in the combination treatment group significantly increased to 53.9 %, compared to 22.4 % in NK cell monotherapy and 17.2 % in TI monotherapy (Fig. 4B). These results indicated that TI promotes apoptosis in AML cells in NK cell-related manner.

### 3.5 The combination therapy of BIBR1532 and NK cells significantly increased caspase 3/7 activity in primary AML and Kg-1a cells compared to monotherapy

**Fig. 5:**
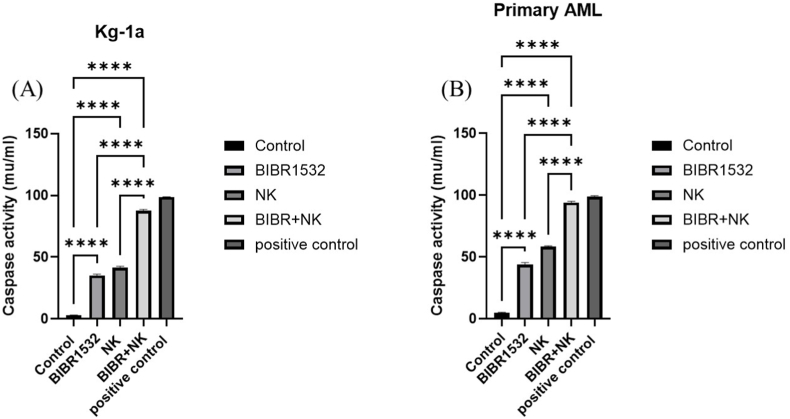
Caspase 3/7 activity of Kg-1a and primary AML cells in different groups. Data represented mean ± standard deviations (n = 3). The P-values are presented as, ∗∗∗∗P < 0.0001.

Caspase activation disrupts cellular structures and initiates apoptosis. Therefore, the activities of caspases 3/7 were analyzed following treatment of primary AML and Kg-1a cells with BIBR1532 and NK cells (Fig. 5). The caspase activity assay demonstrated that primary AML and KG-1a cells treated with combination therapy exhibited significantly higher levels of caspase 3/7 activity compared to monotherapy (Fig. 5A and B).

### 3.6 The combination therapy of BIBR1532 and NK cells significantly changed the expression of Bax and Bcl-2 proteins in primary AML and Kg-1a cells compared to monotherapy

**Fig. 6:**
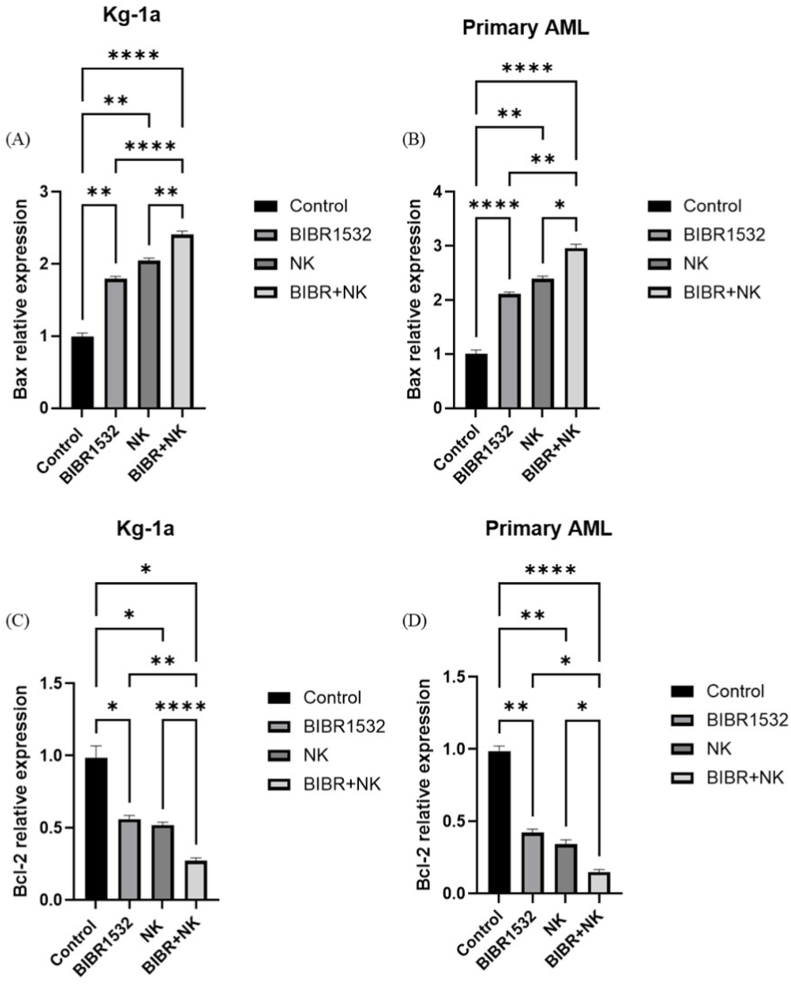
TI/NK cells co-treatment changed expression of Bax and Bcl-2 proteins in AML cells. Relative expression of Bax (A and C) and Bcl-2 (B and D) proteins in primary AML and Kg-1a cells. Data represented mean ± standard deviations (n = 3). The P-values are presented as, ∗p < 0.05, ∗∗p < 0.01, ∗∗∗P ≤ 0.001, and ∗∗∗∗P ≤ 0.0001.

To determine if TI and NK cells in AML can affect the expression of Bax and Bcl-2 proteins, the inhibitory impacts of both single and combined treatments with NK cells and TI were assessed on the protein levels of Kg-1a and primary AML cells using an ELISA assay (Fig. 6). The results showed that the combination therapy with BIBR1532 and NK cells significantly increased the expression of Bax protein in both primary AML and Kg-1a cells in comparison to monotherapy (Fig. 6A–C). Additionally, the combination therapy of BIBR1532 and NK cells led to a substantial reduction in Bcl-2 protein expression in both primary AML and Kg-1a cells compared to the monotherapy (Fig. 6B–D).

### 3.7 hTERT and apoptosis-related gene expression altered after telomerase inhibition/natural killer cell co-treatment

**Fig. 7:**
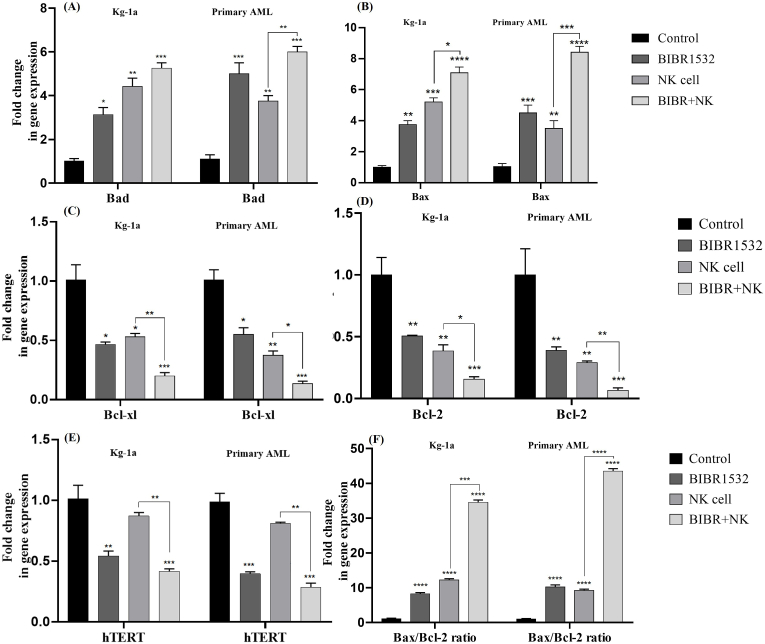
Combination treatment with TI and NK cells altered apoptosis-related and hTERT gene expression in AML cells. The effect of TI/NK cell on transcriptional activity of Bax, Bad, Bcl-2, Bcl-xl, and Bax/Bcl-2 ratio (A, B, C, D, and E). As shown, co-treatment with TI/NK cell up-regulated mRNA levels of pro-apoptotic Bad (A), Bax (B) genes and Bax/Bcl-2 ratio (E) while, it down-regulated the expression levels of Bcl-xl (C) and Bcl-2 (D). The expression levels of hTERT were shown after co-treatment with TI and NK cell using RT-qPCR (F). The significant differences among groups assess by two‐way ANOVA. The values are shown as mean ± (SD) of 3 independent experiments, ∗p < 0.05, ∗∗p < 0.01, ∗∗∗P ≤ 0.001, ∗∗∗∗P ≤ 0.0001.

The combination of TI and NK cells significantly increased the expression of pro-apoptotic genes like Bax, and Bax/Bcl-2 ratio, while down-regulated the expression of anti-apoptotic genes including Bcl-2 and Bcl-xl (Fig. 7A, B, C, D and E). However, changes in Bad pro-apoptotic gene expression were not significant in combination therapy. The combination of TI/NK cell in primary AML cells increased the expression of pro-apoptotic genes (Bax and Bad) and Bax/Bcl-2 ratio and the percentage of anti-apoptotic genes (Bcl-2 and Bcl-xl) decreased significantly compared to single NK cell treated group (Fig. 7A, B, C, D and E). Furthermore, the combination treatment of TI and NK cells significantly reduced hTERT mRNA levels compared to NK cell mono-treatment (Fig. 7F).

## 4 Discussion

Immunotherapy is considered one of the pioneering strategies for enhancing immune responses. Among immune cells, NK cells, as a major component of innate immunity, play a substantial role in developing rapid immune responses [1]. Unlike adaptive immune system cells, NK cells display high cytotoxicity against virus-infected and cancer cells without MHC restriction recognition and previous sensitization [3]. NK-based therapies have been applied in many ongoing clinical trials [23], including AML [24]. According to former studies, NK cells play a key role in the control of AML development and progression [25]. Research conducted on AML patients indicates that patients with NK cells exhibiting enhanced cytolytic activity display improved outcomes and longer remission [25].

However, these therapies, when administered in isolation, demonstrate minimal effects on cancer [12]. Consequently, various strategies have been employed in NK cell-based therapies to maximize therapeutic efficacy against malignancies. This study investigates the co-treatment of NK cells with a telomerase inhibitor, BIBR1532, to enhance NK cell activity. Telomerase is expressed in approximately 85 % of cancer cells, including AML cells [15,26], and its inhibition presents a promising strategy when combined with NK cell therapy. To the best of our knowledge, the effects of telomerase inhibition in conjunction with NK cell treatment have not been previously reported.

Several studies have indicated the TI effect in mono and combination therapies [20,26]. In the previous study, the current researchers reported that TI significantly reduced cell proliferation and increased apoptosis in CD34-positive and negative AML cells [14]. Bashash et al. found that TI increased doxorubicin-induced apoptosis in pre-B Acute Lymphoblastic Leukemia (ALL) cells [27]. The current findings suggest that TI significantly enhances the anti-proliferative effects of NK cells in AML cells. CD107a (LAMP-1) is a functional marker of NK cells that is increased during cytotoxic activity [22]. Interferon (IFN)-γ is a type II IFN that is secreted by activated T and NK cells [28]. IFN-γ supports the cytotoxic responses of NK cells by enhancing the production of perforin and granzyme and augmenting anti-tumor immunity [29].

It has been shown that BIBR1532 increases telomere dysfunction [16]. DNA damage responses and cellular stress lead to activation of NK cells and secretion of IFN-γ [1,30]. A study also showed that targeting hTERT using lentivectors is highly effective at stimulating a wide range of CD8 T-cell immunity, which can be leveraged for cancer immunotherapy [31]. Furthermore, in the previous study, the current authors showed a combination therapy with telomerase inhibitor and NK cells in triple negative breast cancer cells. According to the results TI-induced NK cell cytotoxicity in breast cancer cells [22]. The present results demonstrate that TI enhances CD107a expression and IFN-γ production by NK cells in AML cells.

Adaptive antitumor immunity relies heavily on innate immunity, which serves as the body's first line of defense by recognizing non-self-materials through pattern recognition receptors, including cytosolic DNA sensors. Cancer cells generate cytosolic chromatin fragments and micronuclei that are taken up by antigen presenting cells. This tumor-derived DNA activates the cyclic GMP-AMP (cGAMP) synthase-stimulator of IFN genes (cGAS–STING) pathway, where cytosolic dsDNA binds to cGAS, leading to cGAMP production [32]. This, in turn, triggers conformational changes in STING, activating TANK-binding kinase 1 (TBK1), which phosphorylates IFN regulatory transcription factor 3 (IRF3) and promotes type I IFN expression in cancer cells or antigen presenting cells. Type I IFN signaling is crucial for cross-priming tumor-specific T cells by activating antigen presenting cells, thereby initiating an innate anticancer immune response. Research has demonstrated that targeting telomeres in cancer cells generate damaged DNA fragments, which are taken up by antigen-presenting cells. This process activates the cGAS-STING pathway and boosts adaptive immune responses in preclinical studies [33].

Short-term inhibition of telomerase induces cell cycle arrest and apoptosis [28,34]. BIBR1532 downregulates telomerase activity and induces apoptosis by translational and posttranslational hTERT modifying [35]. On the other hand, NK cells can induce cancer cell death through different mechanisms when encountering malignant cells. For example, these cells can release granzyme and perforin-contained granules into the intercellular space after the interaction with a cancer cell, leading to cancer cell death through caspase-dependent and independent pathways [1]. Moreover, NK cell-induced death in cancer cells may be stimulated through death receptor-mediated apoptosis including TNF-related apoptosis-inducing ligand, Tumor Necrosis Factor (TNF), and Fas ligand [36].

Based on the co-treatment experiment, an enhanced increase in the apoptosis of AML cells was found when NK cells were applied in combination with BIBR1532 suggesting that hTERT can exert its anti-apoptotic function by inhibition of intrinsic apoptosis pathway [37]. NK cells and T cells can release granzymes or utilize death ligands to induce apoptosis in cancer cells [38]. Research has demonstrated that recombinant granzyme B can trigger cytochrome *c* release from the mitochondria of Jurkat cells [39]. Additionally, a study has shown that primary NK cells can induce mitochondrial apoptosis (mtApoptosis). Pre-activated NK cells have been found to cause a loss of mitochondrial outer membrane potential and rapidly prompt cytochrome *c* release from mitochondria; key events that are necessary for mtApoptosis. Furthermore, NK cell treatment quickly activates caspase-3 and triggers the externalization of phosphatidylserine. This treatment also results in the cleavage of BID, as well as caspases 9, 8, and 3, and PARP-1, ultimately leading to membrane blebbing in target cells and the formation of apoptotic bodies [40]. Our study consistent with these findings, showed that TI/NK cell co-treatment enhanced Bax and Bad mRNA expression level and Bax protein while reducing anti-apoptotic genes expression including Bcl-2, Bcl-xl and Bcl-2 protein. Overall, the present results confirm that BIBR1532 and NK cells improve the treatment outcomes in AML patients. These findings provide new insights into hematologic malignancy treatment and hold potential for future clinical applications.

## 5 Conclusion

Altogether, the present investigation indicates that TI promotes NK cell function in Kg-1a and primary AML cells. Furthermore, the combination of TI and NK enhances the anti-proliferative effect and induces apoptosis in AML cells through transcriptional suppression of hTERT, Bcl-2, and Bcl-xl, upregulation of Bax and Bad expression levels, and increasing the Bax/BCl-2 ratio.

## Consent to participate

Informed consent was obtained for all individual participants included in the study.

## Ethics approval

This study was approved by the Ethical Committee of Tabriz University of Medical Sciences (ethic code, IR.TBZMED.REC.1397.394). The study was performed by the Declaration of Helsinki and its later amendments.

## Author contribution

A.R, Kh. D. A, Z. M, and M. T performed all research techniques; A. R wrote the article; H. N. Ch guided the present scientific team, wrote and revised the article. All the authors studied and approved the final manuscript.

## Funding

This work was supported by 10.13039/501100004366Tabriz University of Medical Sciences.

## Declaration of competing interest

The authors declare that they have no known competing financial interests or personal relationships that could have appeared to influence the work reported in this paper.

## Data Availability

Data will be made available on request.
